# Long-Term Prognosis of Non-Ischaemic Myocardial Injury in the Emergency Department: A Propensity Score-Adjusted Study

**DOI:** 10.3390/jcm15218312

**Published:** 2026-10-25

**Authors:** Isabel Jurado-Meléndez, Carla Durà-Roldán, Alfredo Bardají, Isabel Fort, Gilmar Pugnet, Joan Badía, María Llambrich, José Luis Ferreiro

**Affiliations:** 1 Department of Cardiology, Joan XXIII University Hospital, 43005 Tarragona, Spain; isabel.jurado@estudiants.urv.cat (I.J.-M.); carla.dura@estudiants.urv.cat (C.D.-R.); jferreiro.hj23.ics@gencat.cat (J.L.F.); 2 Facultat de Medicina i Ciències de la Salut, University Rovira i Virgili, 43003 Tarragona, Spain; ifgallifa.hj23.ics@gencat.cat (I.F.); gilmair.hj23.ics@gencat.cat (G.P.); 3 Institut de Recerca Biomèdica Catalunya Sud (IRB CatSud), 43005 Tarragona, Spain; joan.badia@irbcatsud.cat (J.B.); maria.llambrich@irbcatsud.cat (M.L.); 4 Clinical Laboratory, Catalan Institute of Health, Camp de Tarragona-Teres de l’Ebre, Joan XXIII University Hospital, 43005 Tarragona, Spain; 5 Emergency Department, Joan XXIII University Hospital, 43005 Tarragona, Spain

**Keywords:** cardiac troponin, non-ischaemic myocardial injury, emergency department, prognosis, mortality, heart failure

## Abstract

**Background/Objectives**: Cardiac troponin measurement in the emergency department frequently detects non-ischaemic myocardial injury. Although this condition accounts for most troponin elevations, its clinical management remains uncertain. This study aims to determine its prevalence and evaluate its prognostic implications at a 5-year follow-up. **Methods**: We conducted a retrospective cohort study of 2715 emergency department patients undergoing systematic cardiac troponin testing, excluding type 1 or 2 acute myocardial infarctions. Patients with non-ischaemic myocardial injury (*n* = 254, 9.4%) were compared to controls (*n* = 2461, 90.6%) using propensity score methods (overlap weighting and 1:1 matching). **Results**: Before matching, patients with non-ischaemic injury were older with higher comorbidities. After overlap weighting, the risks of all-cause mortality (hazard ratio [HR] 1.75, 95% CI 1.45–2.11; *p* < 0.001) and major adverse cardiovascular events (HR 1.78, 95% CI 1.49–2.13; *p* < 0.001) remained significantly higher in the non-ischaemic injury group over a median 5.07-year follow-up. In cause-specific Cox analysis, heart failure readmission rate was increased among survivors (HR 1.64, 95% CI 1.16–2.30; *p* = 0.005); however, when accounting for all-cause mortality as a competing risk, this association attenuated (subdistribution hazard ratio [sHR] 1.27, 95% CI 0.90–1.80; *p* = 0.168). Recurrent myocardial infarction readmissions did not differ between groups (cause-specific HR 1.61, 95% CI 0.70–3.72; *p* = 0.265; Fine–Gray sHR 1.26, 95% CI 0.55–2.91; *p* = 0.596). **Conclusions**: Incidental detection of non-ischaemic myocardial injury in the emergency department independently predicts a substantially poorer long-term prognosis, predominantly driven by all-cause mortality. These findings highlight the clinical importance of recognizing non-ischaemic injury as a marker of high vulnerability requiring close long-term clinical follow-up.

## 1. Introduction

Cardiac troponin (cTn) measurement in the emergency department (ED) is essential for diagnosing type 1 (T1MI) and type 2 (T2MI) myocardial infarction [1,2]. However, systematic or routine cTn testing frequently detects elevated levels without ischaemic criteria [3,4], classified as non-ischaemic myocardial injury (NIMI). NIMI accounts for most troponin elevations—surpassing T1MI and T2MI [5,6,7,8]—and carries a worse prognostic impact, strongly predicting cardiovascular and non-cardiovascular mortality [9,10,11]. Furthermore, recent evidence highlights the prognostic value of routinely measured cardiac troponin beyond acute settings. Resting cardiac troponin levels have been shown to correlate significantly with parameters of subclinical cardiovascular dysfunction, such as markers of subclinical atherosclerosis (e.g., intima-media thickness) and increased vascular stiffness (e.g., pulse wave velocity) [12]. Despite these pathophysiological insights, the long-term prognostic implications of these findings when NIMI is incidentally detected during an acute emergency department visit remain a critical gap in current clinical knowledge.

While clinical practice guidelines provide precise management protocols for T1MI, and emerging evidence offers valuable guidance for T2MI, the clinical management of NIMI remains largely undefined. Despite being the most frequent cause of cTn elevation in the ED, NIMI is insufficiently studied [13]. Current knowledge is largely limited to the fact that these patients present an unfavourable risk profile—often being older individuals with a heavy burden of comorbidities—which portends a poor prognosis [11]. Consequently, given this baseline vulnerability, it is still debated whether their adverse clinical outcomes are driven by underlying systemic disease rather than the myocardial injury itself [5,10].

In light of these considerations, we hypothesize that NIMI detected in the ED is an independent predictor of adverse outcomes, maintaining its detrimental prognostic impact after rigorous adjustment for clinical confounders via propensity score adjustment. Accordingly, the objectives of this study are to determine the true prevalence of NIMI among patients attending the ED, to characterize its clinical profile and baseline characteristics, and to evaluate its prognostic implications over a 5-year follow-up period.

## 2. Materials and Methods

### 2.1. Study Population

We conducted a retrospective cohort study of consecutive adult patients presenting to the Emergency Department (ED) of Joan XXIII University Hospital (Tarragona, Spain) between 22 October 2020, and 11 January 2021, where cTn was systematically measured in all patients undergoing routine blood testing. This systematic testing encompassed both patients for whom cTn was explicitly ordered by the attending physician based on clinical suspicion, as well as those with blood drawn for any other indication where cTn was not requested. In the latter cases, cTn determination was performed on the excess serum retained by the central laboratory, and these specific results were kept strictly blinded to attending clinicians to avoid altering acute patient management.

Inclusion criteria:

- Patients aged ≥18 years presenting to the ED during the study period.
- Having a high-sensitivity cardiac troponin I (hs-cTnI) determination performed as part of systematic ED blood testing.

Exclusion criteria:

- Diagnosis of Type 1 Acute Myocardial Infarction (T1MI), according to the Fourth Universal Definition of Myocardial Infarction.
- Diagnosis of Type 2 Acute Myocardial Infarction (T2MI), defined according to the Saaby criteria.
- Residence outside the hospital’s geographic reference area (to ensure complete follow-up data).
- Incomplete medical records or missing baseline essential data.

Based on clinical data and cTn testing, patients were classified into four groups: absence of cTn elevation (no myocardial injury, control group), T1MI, T2MI, and NIMI. The retrospective adjudication of these diagnoses was performed through a comprehensive review of the electronic medical records by two independent cardiologists, applying the criteria of the Fourth Universal Definition of Myocardial Infarction [13], with T2MI specifically defined by the Saaby criteria [14]. NIMI was defined as cardiac troponin I elevation above the 99th percentile (>47 ng/L) in the absence of clinical, electrocardiographic, or imaging evidence of acute myocardial ischaemia. For the long-term prognostic analysis, patients with T1MI and T2MI, as well as those residing outside our reference area or having incomplete data, were excluded to focus the comparison strictly on the NIMI and control groups.

The study protocol was conducted in accordance with the Declaration of Helsinki and approved by our Institutional Review Board (Ethics Committee of Institut d’Investigació Sanitària Pere Virgili, protocol code: TROPO-URG, ref. CEIM: 195/2020, approved on 6 October 2020). Due to the retrospective design and the use of acellular leftover material, the requirement for individual informed consent was waived by the ethics committee.

### 2.2. Troponin Assay

Cardiac troponin I (cTnI) was measured using a high-sensitivity immunoassay (Siemens Advia Centaur; Siemens Healthineers, Erlangen, Germany). The reference limit for myocardial injury (cTnI positivity) was defined as >47 ng/L (99th percentile, coefficient of variation < 10%).

### 2.3. Clinical Variables Studied

Electronic medical records were reviewed to extract demographics, cardiovascular risk factors, clinical history, vital signs, electrocardiographic findings, and laboratory data. The estimated glomerular filtration rate (eGFR) was calculated using the CKD-EPI formula, and hospital admission status was recorded.

### 2.4. Primary and Secondary Outcomes

The primary outcome was all-cause mortality at 5 years of follow-up. Secondary outcomes included heart failure readmissions, myocardial infarction readmissions, MACE (a composite of the first admission for heart failure, myocardial infarction, or death), and MACE-2 (a composite of the first admission for heart failure or death). MACE-2 was specifically incorporated to provide a targeted assessment of non-atherothrombotic cardiovascular risk (heart failure and mortality), excluding ischaemic coronary events that are less characteristic of non-ischaemic myocardial injury. Follow-up data were exclusively obtained through a comprehensive review of the shared electronic medical records of the Catalan Health System (“Historia Clínica Compartida de Catalunya”—HC3), without conducting phone calls or in-person visits. All outcomes were systematically compared between the NIMI group and the control group across three levels of analysis: unadjusted, age and sex matched adjustment, and propensity score (PS) adjusted analyses.

### 2.5. Statistical Analysis

Categorical variables were presented as frequencies and percentages (n [%]). Continuous variables were expressed as mean and standard deviation (SD) if normally distributed, or as median and interquartile range [P25–P75] for non-normally distributed data. Baseline characteristics between the NIMI and control groups were compared using Pearson’s Chi-square or Fisher’s exact test for categorical variables, Welch’s *t*-test for normally distributed continuous variables, and the Mann–Whitney U test for non-normal data. Missing values in presentation variables (vital signs) were handled using multiple imputation by chained equations (MICE) solely for descriptive completeness [15].

Time-to-event outcomes were evaluated using marginal Cox proportional hazards models with right-censoring. Hazard ratios (HRs) were estimated including exposure as the sole covariate. Comparisons involving fewer than five events in the smaller study arm were omitted. To account for potential confounding, treatment effects were evaluated using propensity score (PS) methods. Confounding variables included in the PS estimation were pre-selected based on clinical relevance and prior evidence regarding chronic baseline risk. To strictly isolate chronic baseline vulnerability from the acute presentation severity, the PS model included exclusively chronic baseline covariates: demographics (age, sex), individual chronic comorbidities, Charlson Comorbidity Index, baseline renal function (eGFR CKD-EPI), haemoglobin, and baseline glucose. Variables reflecting acute physiological distress at emergency arrival (e.g., vital signs) were intentionally excluded. Furthermore, to avoid subjective selection bias and prevent model overfitting from covariate collinearity, high-dimensional regularized regression models (LASSO and Elastic-Net with 10-fold cross-validation) were performed as sensitivity analyses to validate the stability of the selected baseline confounders (Supplementary Material S1.1). To address potential pandemic-related confounding, acute SARS-CoV-2 infection status was systematically recorded during the index presentation. As sensitivity analyses, COVID-19 status was incorporated directly into the propensity score model, and a separate sensitivity analysis was performed after excluding all COVID-19-positive patients with full re-estimation of the propensity scores in the uninfected cohort. Two causal estimands were evaluated for the PS analyses. Primarily, the average treatment effect in the overlap population (ATO) was estimated using overlap weighting (IPTW family) to retain all patients. Secondarily, the average treatment effect on the treated (ATT) was estimated using 1:1 nearest-neighbor matching with a caliper of 0.2 standard deviations of the logit of the PS [16]. To account for the study design, sandwich variance estimators were applied for weighted analyses (ATO), and cluster-robust variance estimators for matched analyses (basic and ATT). For non-fatal secondary outcomes (heart failure and recurrent myocardial infarction readmissions), competing-risk analyses were performed treating all-cause mortality as a competing event. Cumulative incidence functions were estimated using the Aalen–Johansen estimator. Subdistribution hazard ratios (sHR) were calculated using Fine–Gray proportional subdistribution hazards regression with overlap weighting (ATO) and cluster-robust variance estimation. Both cause-specific hazard ratios (reflecting etiological relative risk among surviving individuals) and subdistribution hazard ratios (reflecting absolute population event burden) are reported.

Across all matched and weighted models, covariate balance was verified using the standardized mean difference (SMD), with an |SMD| < 0.1 considered adequate. Detailed technical formulations of these estimators and regularised models are described in the Supplementary Methods (Supplementary Material). E-values were calculated to quantify sensitivity to unmeasured confounding [17]. All statistical tests were two-sided, and a *p*-value < 0.05 was considered statistically significant. All analyses were conducted using R software version 4.4.1 (R Foundation for Statistical Computing, Vienna, Austria) (packages: survival, glmnet, mice, cobalt, MatchIt, survRM2, EValue).

## 3. Results

During the study period, 8525 patients visited the ED, of whom 2902 underwent blood testing. As detailed in the patient selection flowchart (Figure 1), after excluding individuals residing outside our reference area, patients diagnosed with T1MI, T2MI, and those with incomplete medical records, the final analysed cohort comprised 2715 patients. Of these, 2461 (90.6%) formed the control group (absence of NIMI) and 254 (9.4%) presented with NIMI. Thanks to the complete coverage of the centralized electronic health registry within our reference area, complete 5-year follow-up was achieved for all 2715 included patients, and no patients were lost to follow-up.

### 3.1. Baseline Clinical Characteristics

Patients in the NIMI group were significantly older, predominantly male, and had a higher baseline comorbidity burden: higher Charlson index, hypertension, diabetes, previous myocardial infarction, heart failure, and chronic kidney disease. Regarding ED presentation, NIMI patients presented more frequently with dyspnoea, atrial fibrillation, bundle branch blocks, and impaired renal function. Consequently, the NIMI cohort had significantly higher hospital admission and in-hospital mortality rates compared to controls (Table 1). Table 1 lists the primary conditions linked to non-ischaemic troponin release, whereas remaining hospitalizations comprise diverse non-cardiovascular admissions (e.g., traumatology, surgery, neurology, and oncology).

### 3.2. Covariate Balance After Propensity Score Adjustment

As noted in the unadjusted baseline data, significant clinical differences initially existed between patients with NIMI and the control group. Following overlap weighting (ATO) (Table 2) and nearest-neighbour matching (ATT) (Table 3), differences across the chronic baseline covariates included in the models were substantially reduced. Optimal covariate balance was achieved for these chronic clinical and laboratory variables, with standardized mean differences (SMD) falling well below the prespecified threshold of 0.1. As expected, presentation variables reflecting the severity of the acute episode, which were intentionally excluded from the propensity score calculation, maintained higher SMD values. Consequently, the resulting adjusted cohorts were highly comparable regarding their baseline vulnerability, minimizing chronic confounding to reliably isolate the independent prognostic impact of NIMI in the subsequent survival analyses.

### 3.3. Long-Term Clinical Outcomes

The administrative censoring date for the study cohort was 25 March 2026. Median follow-up estimated by reverse Kaplan–Meier was 5.07 years (95% CI 5.06–5.09; IQR 4.85–5.18) in the overall cohort (*n* = 2843) (Supplementary Figure S1). Unadjusted event rates for all evaluated endpoints were significantly higher in the non-ischaemic myocardial injury group compared to the control group (Table 4). This clinical divergence was reflected in a significantly increased unadjusted risk of all-cause mortality, heart failure readmissions, and both composite endpoints (MACE and MACE-2) in the non-ischaemic injury cohort, with unadjusted hazard ratios exceeding 3.50 for all of them (Figure 2). Regarding recurrent myocardial infarction readmissions, initial unadjusted time-to-event analysis indicated an increased risk in the non-ischaemic myocardial injury group (Figure 2). However, after propensity score adjustment for baseline comorbidities and accounting for mortality as a competing risk, this association fully attenuated and lost statistical significance (Supplementary Figure S2). These estimates were characterized by statistical instability due to the low absolute number of recurrent ischaemic events in this subgroup.

### 3.4. Adjusted Survival Analysis

After propensity score adjustment, the hazard ratios for the main clinical endpoints attenuated but remained highly significant, confirming the independent prognostic impact of non-ischaemic myocardial injury (Figure 2). For the primary endpoint of all-cause mortality, non-ischaemic injury remained a strong and independent predictor of death in the propensity score-adjusted model utilizing overlap weighting (adjusted HR 1.75, 95% CI 1.45–2.11; *p* < 0.001) (Figure 2 and Figure 3). Similarly, the adjusted risk of both composite endpoints was identical and remained highly significant in the overlap-weighted analysis (adjusted HR 1.78, 95% CI 1.49–2.13; *p* < 0.001 for both MACE and MACE-2) (Figure 2).

Regarding specific secondary outcomes, the risk of heart failure readmission remained significantly higher in the non-ischaemic injury cohort after adjusting for baseline chronic vulnerability (adjusted HR 1.64, 95% CI 1.16–2.30; *p* = 0.005) (Figure 2 and Figure 3). Conversely, the risk of recurrent myocardial infarction readmissions did not differ significantly between the groups once baseline characteristics were balanced (adjusted HR 1.61, 95% CI 0.70–3.72; *p* = 0.265) (Figure 2 and Figure 3). Cohort sizes, covariate balance, and sensitivity analyses utilizing LASSO and Elastic-Net regressions across all evaluated models are detailed in Supplementary Table S1 and Figure S2, and their corresponding survival curves in Supplementary Figure S3 (Supplementary Material). COVID-19 was recorded at the index visit in 39 of 254 patients with NIMI (15.4%) and 402 of 2461 controls (16.3%). Including COVID-19 status as a covariate in the propensity score model yielded virtually identical hazard ratios across all primary endpoints. Furthermore, after excluding all COVID-19-positive patients and re-estimating the propensity scores in the remaining uninfected cohort (*n* = 2274), the overlap-weighted HRs remained consistent: 1.72 (95% CI 1.40–2.11) for all-cause mortality, 1.75 (95% CI 1.44–2.12) for MACE, and 1.60 (95% CI 1.12–2.29) for heart failure readmissions (Supplementary Table S2). Incorporating NIMI status into baseline risk models significantly improved 5-year discrimination for all-cause mortality (C-index increase\Delta C = 0.0096, *p* < 0.001) and MACE (C-index increase\Delta C = 0.0095, *p* < 0.001), maintaining statistically significant gains when added to comprehensive models incorporating baseline comorbidities (Supplementary Table S3).

### 3.5. Sensitivity Analyses and E-Values

In the 30-day landmark analysis conditioning on early survival, non-ischaemic myocardial injury remained significantly associated with increased long-term risks of all-cause mortality and MACE (both *p* < 0.001), confirming a sustained chronic prognostic disadvantage beyond the initial acute presentation phase (Supplementary Table S4, Supplementary Figures S4–S6). For non-fatal cardiac readmissions evaluated under Fine–Gray competing-risk regression, subdistribution hazard ratios did not reach statistical significance for heart failure (sHR 1.39, *p* = 0.080) or recurrent myocardial infarction (sHR 1.24, *p* = 0.633), reflecting the strong competing effect of all-cause mortality (Supplementary Table S4). High E-values across primary endpoints confirmed substantial robustness against potential unmeasured confounding (Supplementary Table S5).

## 4. Discussion

This study demonstrates that NIMI is a frequent finding when cTn is systematically evaluated in the ED. Patients presenting with this condition exhibit a high-risk clinical profile and a highly adverse long-term prognosis. Crucially, our propensity-adjusted analysis indicates that NIMI is not merely a bystander, but an independent predictor associated with reduced patient survival.

Our findings align with the limited literature on systematic cTn testing. Lee et al. reported a 12.4% rate of cTn elevations, with NIMI accounting for 11.9%, while T1MI (0.2%) and T2MI (0.3%) were rare [4]. Similarly, Shah et al. found a 13.7% elevation rate, with 10.8% classified as NIMI [18]. Our observed NIMI prevalence of 9.4% is highly consistent with these trials. Furthermore, the clinical phenotype of our NIMI cohort—characterized by older age, renal impairment, and multi-morbidity—strictly mirrors the high-risk profiles described by Lee et al. (mean age 78 years, higher comorbidities).

The clinical utility of routine, unselected cTn testing in the ED has been widely debated, given its significant clinical and statistical limitations [3]. From a statistical perspective, applying standard 99th percentile thresholds to unselected cohorts with a low pre-test probability of acute coronary syndrome precipitously degrades the assay’s positive predictive value [19]. This practice drives widespread overdiagnosis, with up to 14% of general admissions—and considerably more among those over 70 years of age—exhibiting elevated levels without underlying coronary atherothrombosis [20]. Consequently, routine testing disproportionately increases the detection of NIMI and T2MI. Because therapeutic interventions for these entities must target the underlying systemic illness rather than coronary pathology, incidental elevations frequently trigger a deleterious diagnostic cascade. This cascade exposes patients to unnecessary intensive care admissions, invasive procedures, and unwarranted iatrogenic risks from empirical antithrombotic therapies that offer no ischaemic benefit [21].

Infections and heart failure were the predominant conditions associated with NIMI. Pathophysiologically, cTn release in heart failure is driven by wall stretch, neurohormonal activation, and apoptosis, while in infections it is mediated by cytokine cardiotoxicity, microvascular dysfunction, and metabolic demand. Rather than affecting pristine hearts, these acute stressors unmask subclinical structural alterations—like left ventricular hypertrophy, diffuse fibrosis, or endothelial dysfunction—which lower the injury threshold in a pre-existing vulnerable myocardium [21,22]. This concept aligns with our clinical follow-up: high rates of heart failure readmissions and mortality, but no increased risk of recurrent myocardial infarction. The absence of recurrent MI suggests that adverse events stem from structural myocardial disease rather than acute coronary atherothrombosis, as reflected by the near-identity of our MACE and MACE-2 estimates, where composite endpoints were heavily dominated by mortality [23,24,25].

A key methodological insight of our study is the impact of competing mortality on non-fatal cardiac outcomes. While cause-specific hazard models indicated a 64% increase in the rate of heart failure readmission among surviving patients with NIMI, accounting for death as a competing risk via Fine–Gray regression attenuated this relationship (sHR 1.27, *p* = 0.168). This finding highlights that the massive early and overall mortality observed in the NIMI cohort (61.0% at 5 years) acts as a strong competing event, preventing a substantial proportion of patients from experiencing subsequent readmissions. Consequently, while NIMI identifies a population at heightened physiological susceptibility for heart failure, the primary clinical determinant of their long-term course remains overwhelming all-cause mortality.

A major strength of this study is the systematic evaluation of cTn in an unselected cohort of consecutive patients. By analysing all patients undergoing blood testing in the ED regardless of clinical indication, we effectively minimized selection bias. Furthermore, the final cohort has very few exclusions due to missing data or loss to follow-up, and it benefits from a very long-term follow-up period (median of 5.07 years), providing a consistent time window to evaluate the clinical trajectory of these patients. The use of propensity score methods (matching and overlap weighting) adds great robustness by effectively minimizing baseline imbalances.

Nevertheless, the study has limitations that must be acknowledged. As a single-centre analysis, its findings may not be easily generalizable to populations in other healthcare settings. Although the overall sample is large, the size of the specific cohort with NIMI is relatively limited compared to the broad control group. Additionally, we rely exclusively on variables collected during the ED episode; for instance, we lack echocardiographic studies that would allow us to document the exact degree of underlying structural heart disease. The design does not account for the difference between acute and chronic myocardial injury, which could have distinct prognostic implications. The low number of secondary ischaemic events—specifically recurrent myocardial infarction readmissions—limits statistical power and introduces instability for this endpoint, as reflected by wide confidence intervals across matched models. Finally, recruitment occurred during a major COVID-19 surge (late 2020 to early 2021), with SARS-CoV-2 infection present in approximately 15–16% of presentations. Although sensitivity analyses excluding COVID-19 cases confirmed that the long-term prognostic impact of NIMI remained consistent among uninfected patients, potential unmeasured pandemic-related factors cannot be completely excluded. Therefore, while these findings are reassuring, caution is advised when extrapolating results to non-pandemic emergency settings, and confirmation in post-pandemic cohorts is warranted.

## 5. Conclusions

In emergency department patients undergoing cardiac troponin testing, non-ischaemic myocardial injury is independently associated with a marked increase in long-term all-cause mortality and MACE after robust propensity score adjustment. Identifying this condition provides an essential risk-stratification signal to identify a vulnerable patient population that warrants careful clinical monitoring and optimization of underlying medical comorbidities.

## Figures and Tables

**Figure 1:**
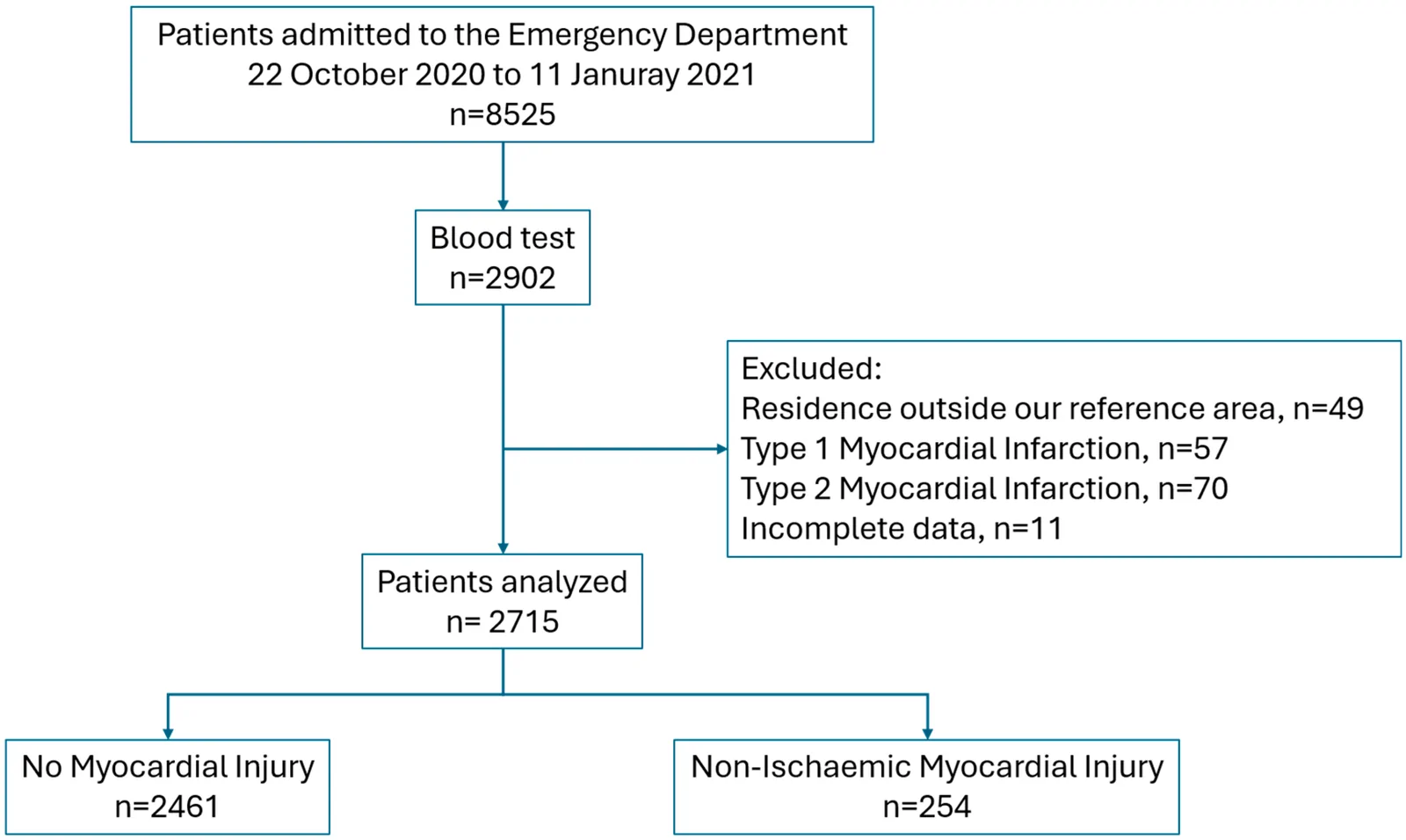
Patient flow diagram. Schematic representation of study patient screening, exclusion criteria, and final cohort stratification based on myocardial injury status. NIMI, non-ischaemic myocardial injury.

**Figure 2:**
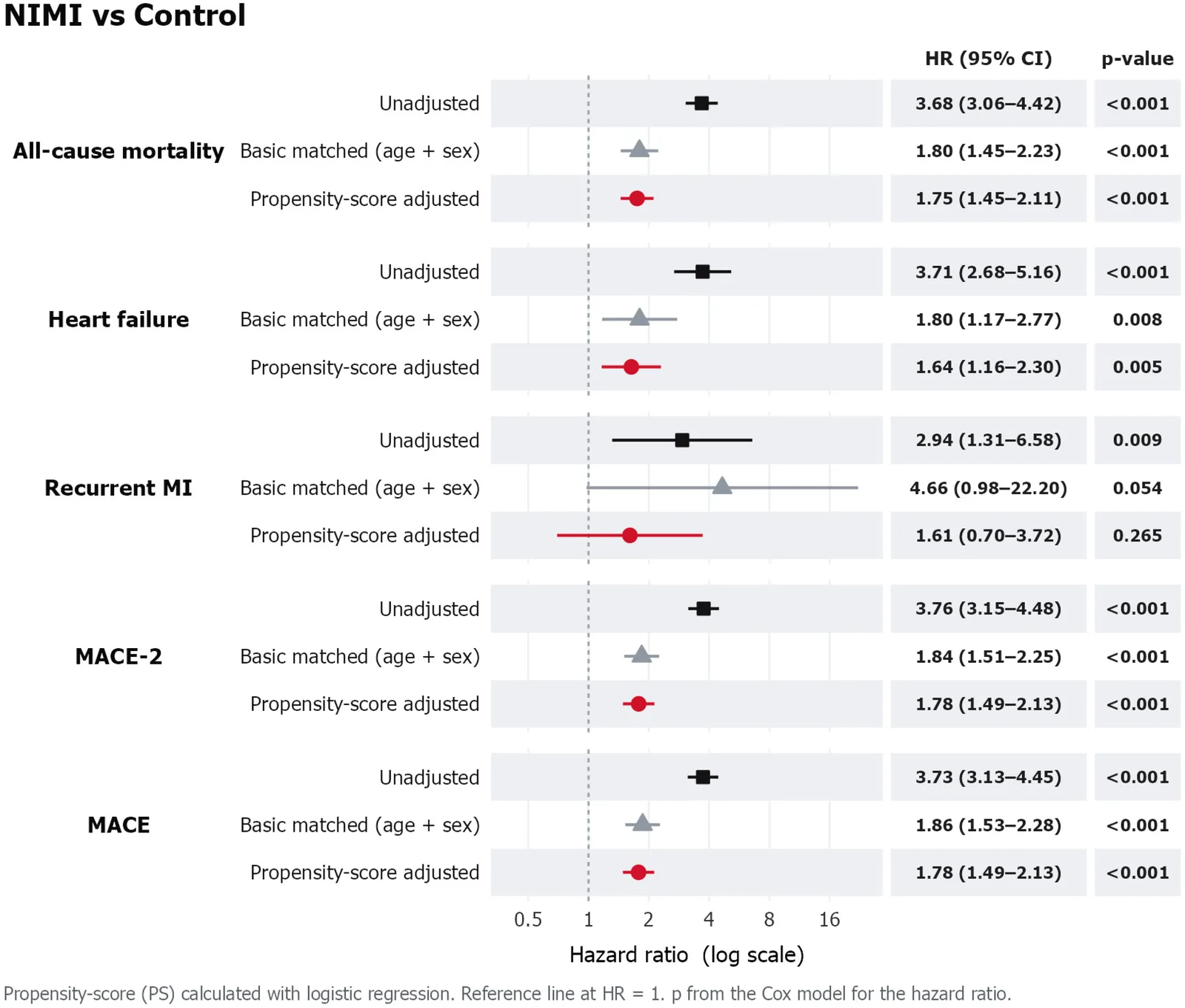
Forest plot of unadjusted and adjusted hazard ratios for five-year clinical outcomes. Hazard ratios and 95% confidence intervals are shown comparing patients with non-ischaemic myocardial injury (NIMI) to the control group across three analytical models: unadjusted (black squares), basic matched for age and sex (grey triangles), and propensity score-adjusted using overlap weighting (red circles). Cause-specific Cox proportional hazards models are reported. For non-fatal secondary endpoints (heart failure and recurrent myocardial infarction readmissions), subdistribution hazard ratios (sHR) accounting for all-cause mortality as a competing risk are presented in Figure 3 and Supplementary Figure S2. The dashed vertical line represents a hazard ratio of 1.0 (null hypothesis/no difference in risk). ATO, average treatment effect in the overlap population (overlap weighting); CI, confidence interval; HR, hazard ratio; MACE, major adverse cardiovascular events (composite of death, heart failure, or recurrent myocardial infarction readmission); MACE-2, composite of death or heart failure readmission; MI, myocardial infarction; NIMI, non-ischaemic myocardial injury; PS, propensity score; sHR, subdistribution hazard ratio.

**Figure 3:**
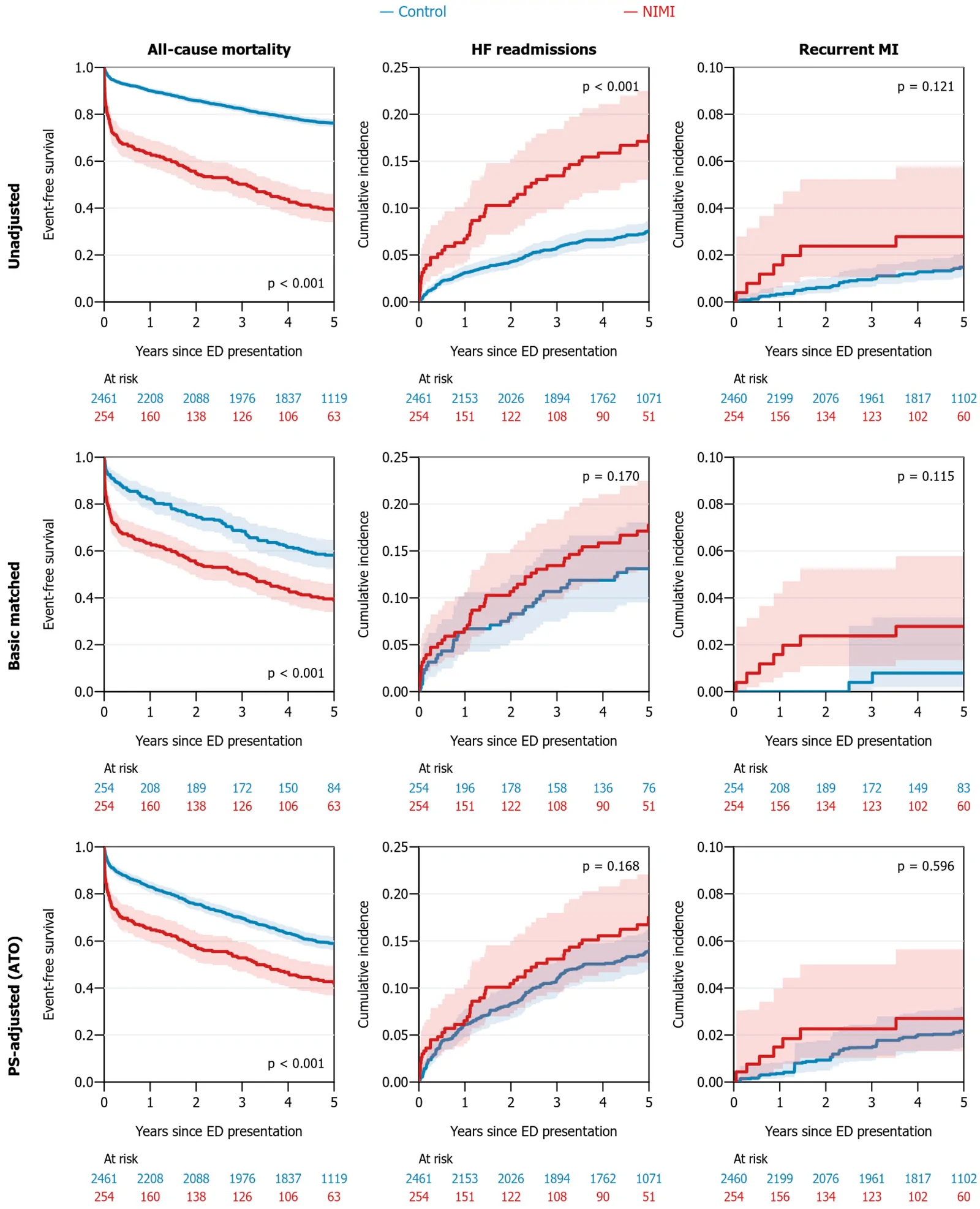
Survival and cumulative incidence over five years of follow-up. Curves compare NIMI (red) with the control group (blue). Columns show all-cause mortality, heart failure readmissions and recurrent myocardial infarction readmissions; rows show unadjusted, basic matched using age and sex, and propensity score-adjusted analyses using overlap weighting. Mortality is shown as Kaplan–Meier survival. First readmissions are shown as Aalen–Johansen cumulative incidence, with death as a competing event. Shading represents 95% confidence intervals. Numbers at risk are unweighted. The *p*-values are from Cox models for mortality and Fine–Gray models for readmissions, using the full recorded follow-up. ATO, average treatment effect in the overlap population; NIMI, non-ischaemic myocardial injury; PS, propensity score.

**Table 1:** Baseline and clinical characteristics of patients with non-ischaemic myocardial injury versus the control group before propensity score matching.

Characteristic	Unadjusted			
	Control (*n* = 2461)	NIMI (*n* = 254)	*p*-Value	SMD
**Demographics**				
Mean age, years (SD)	60.4 (19.1)	72.7 (16.3)	<0.001	0.692
Female, *n* (%)	1153 (46.9%)	109 (42.9%)	0.258	−0.079
**Comorbidities**				
Previous myocardial infarction	151 (6.1%)	30 (11.8%)	<0.001	0.200
Chronic heart failure	101 (4.1%)	31 (12.2%)	<0.001	0.299
Peripheral vascular disease	121 (4.9%)	26 (10.2%)	<0.001	0.202
Previous stroke	130 (5.3%)	24 (9.4%)	0.010	0.160
Chronic respiratory diseases	288 (11.7%)	46 (18.1%)	0.004	0.181
Chronic kidney disease	194 (7.9%)	59 (23.2%)	<0.001	0.433
High Charlson index	363 (14.8%)	85 (33.5%)	<0.001	0.448
**Risk factors**				
Hypertension	1058 (43.0%)	174 (68.5%)	<0.001	0.531
Smoking	313 (12.7%)	27 (10.6%)	0.391	−0.065
Dyslipidaemia	728 (29.7%)	122 (48.2%)	<0.001	0.388
Diabetes mellitus	486 (19.7%)	69 (27.2%)	0.007	0.176
**Symptoms**				
Chest pain	228 (9.3%)	28 (11.0%)	0.423	0.058
Dyspnoea	447 (18.2%)	80 (31.5%)	<0.001	0.312
Syncope	70 (2.8%)	13 (5.1%)	0.070	0.116
Other symptoms	2044 (83.1%)	186 (73.2%)	<0.001	−0.239
**ECG**				
LBBB/RBBB	84 (10.0%)	24 (18.3%)	0.007	0.242
Atrial fibrillation	130 (15.4%)	38 (29.0%)	<0.001	0.331
Pacemaker	23 (2.7%)	5 (3.8%)	0.412	0.061
**Vital signs**				
Heart rate (bpm) †	84.0 [73.0–96.0]	89.0 [72.0–100.8]	0.038	0.184
Systolic BP (mmHg)	136.2 (24.0)	134.2 (28.5)	0.344	−0.074
Oxygen saturation (%) †	98.0 [97.0–100.0]	97.0 [94.0–99.0]	<0.001	−0.441
**Laboratory**				
Glucose (mg/dL) †	109.5 [96.0–135.0]	129.0 [107.0–175.2]	<0.001	0.340
eGFR CKD-EPI (mL/min)	82.3 (28.7)	56.6 (31.8)	<0.001	−0.847
Haemoglobin (g/dL)	12.9 (2.0)	12.8 (1.8)	0.216	−0.077
**In-hospital management**				
Hospital admission	746 (30.3%)	174 (68.5%)	<0.001	0.827
In-hospital death	73 (3.0%)	55 (21.7%)	<0.001	0.593
**Main discharge diagnosis**				
Infection	208 (8.5%)	30 (11.8%)	0.092	0.111
Heart failure	71 (2.9%)	20 (7.9%)	<0.001	0.222
Sepsis	5 (0.2%)	14 (5.5%)	<0.001	0.323
Renal failure	17 (0.7%)	13 (5.1%)	<0.001	0.266
Hypertensive crisis	23 (0.9%)	10 (3.9%)	<0.001	0.196
Tachyarrhythmia	39 (1.6%)	12 (4.7%)	0.002	0.180
Pulmonary embolism	13 (0.5%)	7 (2.8%)	0.002	0.176
Stroke	87 (3.5%)	11 (4.3%)	0.638	0.041

Values are presented as *n* (%) for categorical variables, mean (SD) for normally distributed continuous variables, and median [interquartile range] for variables marked with † (non-normally distributed continuous variables). *p*-values are calculated using Pearson’s chi-square or Fisher’s exact test for categorical variables, Welch’s *t*-test for normally distributed continuous variables, and the Mann–Whitney U test for non-normally distributed continuous variables. BP, blood pressure; bpm, beats per minute; CKD-EPI, Chronic Kidney Disease Epidemiology Collaboration; eGFR, estimated glomerular filtration rate; LBBB, left bundle branch block; NIMI, non-ischaemic myocardial injury; RBBB, right bundle branch block; SD, standard deviation; SMD, standardized mean difference (an absolute value < 0.10 indicates adequate balance between groups).

**Table 2:** Baseline and clinical characteristics of patients with non-ischaemic myocardial injury versus the control group after propensity score adjustment using overlap weighting.

Characteristic	Control (*n* = 2461)	NIMI (*n* = 254)	*p*-Value	SMD
**Demographics**				
Age (years)	71.4 (15.5)	71.4 (16.8)	1.000	0.000
Female sex	43.2%	43.2%	1.000	−0.000
**Comorbidities**				
Previous myocardial infarction	11.2%	11.2%	1.000	0.000
Chronic heart failure	10.7%	10.7%	1.000	0.000
Peripheral vascular disease	9.2%	9.2%	1.000	0.000
Previous stroke	9.1%	9.1%	1.000	0.000
Chronic respiratory diseases	17.3%	17.3%	1.000	0.000
Chronic kidney disease	19.7%	19.7%	1.000	0.000
High Charlson index	29.0%	29.9%	0.776	0.020
**Risk factors**				
Hypertension	65.5%	65.5%	1.000	0.000
Smoking	10.9%	10.9%	1.000	0.000
Dyslipidaemia	41.8%	46.1%	0.211	0.087
Diabetes mellitus	26.6%	26.6%	1.000	0.000
**Symptoms**				
Chest pain	8.5%	11.7%	0.107	0.106
Dyspnoea	23.0%	30.3%	0.014	0.166
Syncope	2.9%	5.5%	0.034	0.131
Other symptoms	80.0%	73.5%	0.021	−0.156
**ECG**				
LBBB/RBBB	12.6%	18.2%	0.109	0.154
Atrial fibrillation	22.9%	26.9%	0.351	0.091
Pacemaker	4.0%	3.7%	0.851	−0.019
**Vital signs**				
Heart rate (bpm) †	82.0 [71.0–94.0]	89.0 [72.0–101.0]	0.003	0.282
Systolic BP (mmHg)	138.8 (25.5)	134.5 (28.6)	0.047	−0.160
Oxygen saturation (%) †	98.0 [96.0–99.0]	97.0 [94.0–99.0]	0.002	−0.260
**Laboratory**				
Glucose (mg/dL) †	120.0 [101.0–159.6]	127.5 [105.0–171.7]	0.054	−0.001
eGFR CKD-EPI (mL/min)	60.5 (28.2)	60.6 (31.8)	0.981	0.002
Haemoglobin (g/dL)	12.8 (2.2)	12.8 (1.8)	0.996	0.000
**In-hospital management**				
Hospital admission	36.0%	67.9%	<0.001	0.673
In-hospital death	5.5%	20.6%	<0.001	0.458
**Main discharge diagnosis**				
Infection	8.0%	11.9%	0.042	0.133
Heart failure	5.8%	7.6%	0.289	0.072
Sepsis	0.5%	5.4%	<0.001	0.297
Renal failure	1.7%	4.4%	0.009	0.161
Hypertensive crisis	1.2%	4.2%	0.001	0.188
Tachyarrhythmia	2.0%	4.6%	0.013	0.148
Pulmonary embolism	0.5%	2.9%	<0.001	0.180
Stroke	5.3%	4.9%	0.804	−0.018

Values are presented as weighted percentages for categorical variables, and as weighted means (standard deviation) or weighted medians [interquartile range] for continuous variables marked with † (non-normally distributed continuous variables). *p*-values are design-based (calculated using the survey package in R) utilizing the weighted Student’s *t*-test for normally distributed continuous variables, the weighted rank-sum test for non-normally distributed continuous variables, and the Rao-Scott chi-square test for categorical variables. Weighted standardized mean difference is the primary metric used to assess covariate balance, where an absolute value < 0.10 indicates adequate balance between groups. ATO, average treatment effect in the overlap population (overlap weighting); BP, blood pressure; bpm, beats per minute; CKD-EPI, Chronic Kidney Disease Epidemiology Collaboration; eGFR, estimated glomerular filtration rate; LBBB, left bundle branch block; NIMI, non-ischaemic myocardial injury; PS, propensity score; RBBB, right bundle branch block; SD, standard deviation; SMD, standardized mean difference.

**Table 3:** Baseline and clinical characteristics of patients with non-ischaemic myocardial injury versus the control group after 1:1 propensity score matching.

Characteristic	PS Matched			
	Control (*n* = 251)	NIMI (*n* = 251)	*p*-Value	SMD
**Demographics**				
Mean age, years (SD)	70.9 (17.5)	72.7 (16.4)	0.237	0.106
Female, *n* (%)	111 (44.2%)	109 (43.4%)	0.928	−0.016
**Comorbidities**				
Previous myocardial infarction	35 (13.9%)	30 (12.0%)	0.595	−0.059
Chronic heart failure	30 (12.0%)	31 (12.4%)	1.000	0.012
Peripheral vascular disease	27 (10.8%)	25 (10.0%)	0.884	−0.026
Previous stroke	23 (9.2%)	23 (9.2%)	1.000	0.000
Chronic respiratory diseases	56 (22.3%)	45 (17.9%)	0.266	−0.109
Chronic kidney disease	65 (25.9%)	57 (22.7%)	0.466	−0.074
High Charlson index	86 (34.3%)	82 (32.7%)	0.777	−0.034
**Risk factors**				
Hypertension	172 (68.5%)	171 (68.1%)	1.000	−0.009
Smoking	26 (10.4%)	27 (10.8%)	1.000	0.013
Dyslipidaemia	111 (44.2%)	121 (48.4%)	0.397	0.084
Diabetes mellitus	72 (28.7%)	66 (26.3%)	0.617	−0.054
**Symptoms**				
Chest pain	23 (9.2%)	28 (11.2%)	0.555	0.066
Dyspnoea	65 (25.9%)	79 (31.5%)	0.200	0.124
Syncope	5 (2.0%)	13 (5.2%)	0.093	0.172
Other symptoms	199 (79.3%)	183 (72.9%)	0.116	−0.150
**ECG**				
LBBB/RBBB	9 (8.9%)	24 (18.3%)	0.065	0.277
Atrial fibrillation	30 (29.7%)	38 (29.0%)	1.000	−0.015
Pacemaker	7 (6.9%)	5 (3.8%)	0.446	−0.138
**Vital signs**				
Heart rate (bpm) †	83.0 [71.0–94.0]	89.0 [72.0–100.0]	0.012	0.311
Systolic BP (mmHg)	137.0 (27.0)	134.1 (28.6)	0.292	−0.106
Oxygen saturation (%) †	98.0 [96.0–99.0]	97.0 [94.0–99.0]	0.017	−0.263
**Laboratory**				
Glucose (mg/dL) †	121.0 [102.0–164.0]	129.0 [107.0–172.0]	0.284	−0.078
eGFR CKD-EPI (mL/min)	56.9 (30.4)	57.1 (31.7)	0.954	0.005
Haemoglobin (g/dL)	12.9 (2.3)	12.8 (1.8)	0.586	−0.049
**In-hospital management**				
Hospital admission	94 (37.5%)	172 (68.5%)	<0.001	0.655
In-hospital death	13 (5.2%)	54 (21.5%)	<0.001	0.495
**Main discharge diagnosis**				
Infection	29 (11.6%)	30 (12.0%)	1.000	0.012
Heart failure	19 (7.6%)	20 (8.0%)	1.000	0.015
Sepsis	1 (0.4%)	14 (5.6%)	0.002	0.308
Renal failure	6 (2.4%)	13 (5.2%)	0.161	0.147
Hypertensive crisis	2 (0.8%)	10 (4.0%)	0.041	0.210
Tachyarrhythmia	4 (1.6%)	12 (4.8%)	0.075	0.182
Pulmonary embolism	2 (0.8%)	7 (2.8%)	0.176	0.151
Stroke	11 (4.4%)	11 (4.4%)	1.000	0.000

Values are presented as *n* (%) for categorical variables, mean (SD) for normally distributed continuous variables, and median [interquartile range] for variables marked with † (non-normally distributed continuous variables). *p*-values are calculated using Pearson’s chi-square or Fisher’s exact test for categorical variables, Welch’s *t*-test for normally distributed continuous variables, and the Mann–Whitney U test for non-normally distributed continuous variables. BP, blood pressure; bpm, beats per minute; CKD-EPI, Chronic Kidney Disease Epidemiology Collaboration; eGFR, estimated glomerular filtration rate; LBBB, left bundle branch block; NIMI, non-ischaemic myocardial injury; RBBB, right bundle branch block; SD, standard deviation; SMD, standardized mean difference (an absolute value < 0.10 indicates adequate balance between groups).

**Table 4:** Five-year follow-up events and clinical outcomes of patients with non-ischaemic myocardial injury versus the control group before propensity score matching.

Follow-Up Events	Unadjusted			
	Control (*n* = 2461)	NIMI (*n* = 254)	*p*-Value	SMD
All-cause mortality	585 (23.8%)	155 (61.0%)	<0.001	0.814
Heart failure	181 (7.4%)	44 (17.3%)	<0.001	0.307
Recurrent myocardial infarction	37 (1.5%)	7 (2.8%)	0.183	0.087
MACE	678 (27.5%)	172 (67.7%)	<0.001	0.878
MACE-2	660 (26.8%)	170 (66.9%)	<0.001	0.878

Values are presented as *n* (%) representing the cumulative number of patients experiencing the event and the corresponding raw percentage. *p*-values are calculated using Pearson’s chi-square test or Fisher’s exact test, as appropriate. MACE, major adverse cardiovascular events (defined as a composite of all-cause mortality, first readmission for heart failure, or first readmission for recurrent myocardial infarction); MACE-2, composite of all-cause mortality or first readmission for heart failure; MI, myocardial infarction; NIMI, non-ischaemic myocardial injury; SMD, standardized mean difference.

## Data Availability

The data underlying this article will be shared on reasonable request to the corresponding author.

## Supplementary Materials

The following supporting information can be downloaded at: https://www.mdpi.com/article/10.3390/jcm15218312/s1, Section S1. Supplementary Methods: S1.1. High-Dimensional Variable Selection (LASSO & Elastic-Net); S1.2. Propensity Score Causal Estimands (ATO & ATT); S1.3. Competing-Risk Analyses (Fine–Gray & Aalen–Johansen CIF); S1.4. Incremental Prognostic Value and Model Discrimination (Harrell’s C-index); S1.5. Sensitivity Analyses (30-Day Landmark, E-Values, and COVID-19 Exclusion). Section S2. Supplementary Tables: Supplementary Table S1: Sample size, cohort composition, and covariate balance status across all eight propensity score models evaluated; Supplementary Table S2: Sensitivity analysis excluding patients with confirmed COVID-19 infection; Supplementary Table S3: Incremental prognostic value of non-ischaemic myocardial injury over baseline risk models; Supplementary Table S4: 30-day landmark sensitivity analysis clinical outcomes (cause-specific Cox HRs and Fine–Gray sHRs); Supplementary Table S5: E-values for overall 5-year follow-up and 30-day landmark analyses across clinical outcomes. Section S3. Supplementary Figures: Supplementary Figure S1: Reverse Kaplan–Meier curve for overall cohort follow-up duration; Supplementary Figure S2: Fine–Gray subdistribution hazard ratios (sHR) forest plot for non-fatal cardiac readmissions; Supplementary Figure S3: Kaplan–Meier 5-year mortality survival curves across eight propensity score models; Supplementary Figure S4: 30-day landmark survival and cumulative incidence 3 × 3 grid; Supplementary Figure S5: 30-day landmark cause-specific Cox hazard ratios forest plot; Supplementary Figure S6: 30-day landmark Fine–Gray subdistribution hazard ratios forest plot.

## Abbreviations

The following abbreviations are used in this manuscript:

ATO	Average treatment effect in the overlap population
ATT	Average treatment effect on the treated
BP	Blood pressure
bpm	Beats per minute
CI	Confidence interval
CKD-EPI	Chronic Kidney Disease Epidemiology Collaboration
cTn	Cardiac troponin
cTnI	Cardiac troponin I
ED	Emergency department
eGFR	Estimated glomerular filtration rate
HR	Hazard ratio
IRB CatSud	Institut de Recerca Biomèdica Catalunya Sud
LBBB	Left bundle branch block
MACE	Major adverse cardiovascular events
MACE-2	Composite of all-cause mortality or first readmission for heart failure
MI	Myocardial infarction
MICE	Multiple imputation by chained equations
NIMI	Non-ischaemic myocardial injury
PS	Propensity score
RBBB	Right bundle branch block
SD	Standard deviation
SMD	Standardized mean difference
T1MI	Type 1 myocardial infarction
T2MI	Type 2 myocardial infarction
