# Supplementary material for: Long-Term Prognosis of Non-Ischaemic Myocardial Injury in the Emergency Department: A Propensity Score-Adjusted Study
Source: J Clin Med. 2026 Oct 25;15(21):8312. doi: 10.3390/jcm15218312 (PMC13764488; doi:10.3390/jcm15218312)
Supplement: Supplementary file 1 [file jcm-15-08312-s001.zip › jcm-4590407-supplementary.pdf]

SUPPLEMENTARY MATERIAL

Long-term prognosis of non-ischaemic myocardial injury in the emergency department: a propensity score-adjusted study

Isabel Jurado-Meléndez, Carla Durà-Roldán, Alfredo Bardají, Isabel Fort, Gilmar Pugnet, Joan Badía, María Llambrich, José Luis Ferreiro

Section S1. Supplementary Methods 2
Supplementary Methods S1.1. High-Dimensional Variable Selection (LASSO & Elastic-Net)........ 2
Supplementary Methods S1.2. Propensity Score Causal Estimands (ATO & ATT) 2
Supplementary Methods S1.3. Competing-Risk Analyses (Fine–Gray & Aalen–Johansen CIF) 3
Supplementary Methods S1.4. Incremental Prognostic Value and Model Discrimination (Harrell's C-index) 3
Supplementary Methods S1.5. Sensitivity Analyses (30-Day Landmark, E-Values, and COVID-19 Exclusion) ... 4
Section S2. Supplementary Tables 6
Supplementary Table S1. Sample size, cohort composition, and covariate balance status across all eight propensity score models evaluated. 6
Supplementary Table S2. Sensitivity analysis excluding patients with confirmed COVID-19 infection. 7
Supplementary Table S3. Incremental prognostic value of non-ischaemic myocardial injury over baseline risk models. 8
Supplementary Table S4. 30-day landmark sensitivity analysis clinical outcomes (cause-specific Cox HRs and Fine–Gray sHRs)........ 9
Supplementary Table S5. E-values for overall 5-year follow-up and 30-day landmark analyses across clinical outcomes........ 10
Section S3. Supplementary Figures........ 11
Supplementary Figure S1. Reverse Kaplan–Meier curve for overall cohort follow-up duration. 11
Supplementary Figure S2. Fine–Gray subdistribution hazard ratios (sHR) forest plot for non-fatal cardiac readmissions 12
Supplementary Figure S3. Kaplan–Meier 5-year mortality survival curves across eight propensity score models. 13
Supplementary Figure S4. 30-day landmark survival and cumulative incidence 3x3 grid. 15
Supplementary Figure S5. 30-day landmark cause-specific Cox hazard ratios forest plot. 16
Supplementary Figure S6. 30-day landmark Fine–Gray subdistribution hazard ratios forest plot. 17

Section S1. Supplementary Methods

Supplementary Methods S1.1. High-Dimensional Variable Selection (LASSO & Elastic-Net)

To evaluate the robustness of our primary findings and address potential model overfitting during propensity score estimation, we performed high-dimensional variable selection using regularized regressions as sensitivity analyses. Least Absolute Shrinkage and Selection Operator (LASSO) regression (L1 regularization) and Elastic-Net regression (combining L1 and L2 penalties) were implemented to identify the most parsimonious and stable set of chronic baseline predictors of non-ischaemic myocardial injury.

For both regularized models, the tuning parameters (alpha and lambda) were selected via 10-fold cross-validation minimizing the binomial deviance. These machine-learning-driven regression techniques help prevent overfitting arising from collinearity and a high number of covariates, particularly in clinical settings where multiple chronic comorbidities often coexist. This approach ensured that the probability of belonging to the non-ischaemic myocardial injury group was modeled strictly on independent chronic baseline characteristics, isolating baseline vulnerability from the acute physiological presentation.

Supplementary Methods S1.2. Propensity Score Causal Estimands (ATO & ATT)

Using the estimated propensity scores, we calculated two distinct causal estimands to evaluate the treatment effects (treating non-ischaemic myocardial injury as the exposure):

- **Average Treatment Effect in the Overlap Population (ATO):** Calculated using overlap weighting, where each patient is weighted by the probability of receiving the opposite treatment ($w = e$ for control group and $w = 1-e$ for non-ischaemic injury group, where e is the propensity score). This weighting scheme focuses the analysis on the subpopulation with the greatest empirical overlap in covariates (i.e., patients in clinical equipoise), achieves exact mean balance of all covariates included in a logistic model in finite samples, and completely eliminates the variance inflation caused by extreme propensity scores near 0 or 1.
- **Average Treatment Effect on the Treated (ATT):** Calculated using 1:1 nearest-neighbor matching with a caliper of 0.2 standard deviations of the logit of the propensity score. This approach compares patients with non-ischaemic injury to a highly comparable control cohort, representing the prognostic impact of injury specifically within the clinical population that actually experiences it.

Covariate balance across all models was verified using the standardized mean difference (SMD), where an absolute value $|SMD| < 0.10$ was considered indicative of adequate balance. All survival analyses were performed using marginal Cox proportional hazards models, applying cluster-robust variance

estimators for matched analyses (basic matching and ATT) and sandwich variance estimators for weighted analyses (ATO).

Supplementary Methods S1.3. Competing-Risk Analyses (Fine–Gray & Aalen–Johansen CIF)

In long-term survival studies where overall mortality is high, non-fatal secondary endpoints—such as readmissions for heart failure or recurrent myocardial infarction—are subject to competing risks. Standard cause-specific Cox proportional hazards models treat death as a censoring event. While cause-specific hazard ratios (HR) estimate the relative risk of the non-fatal event strictly among surviving individuals (an etiological perspective), this approach can overestimate the cumulative incidence of non-fatal events in populations with high mortality, as patients who die are no longer at risk of experiencing subsequent readmissions.

To evaluate the absolute population-level event burden and account for all-cause mortality as a competing event, competing-risk analyses were conducted for all non-fatal readmissions:

- Aalen–Johansen Cumulative Incidence Functions (CIF): Cumulative incidence curves for first heart failure readmission and first recurrent myocardial infarction readmission were estimated using the non-parametric Aalen–Johansen estimator, treating all-cause mortality as a competing risk. Unweighted, basic matched, and overlap-weighted (ATO) cumulative incidence curves with 95% confidence intervals were generated.
- Fine–Gray Proportional Subdistribution Hazards Regression: To obtain adjusted hazard ratios that directly relate to the cumulative incidence function, Fine–Gray subdistribution hazard models were fitted. Subdistribution hazard ratios (sHR) and their corresponding 95% confidence intervals were estimated across three levels of adjustment (unadjusted, basic matched for age and sex, and propensity score-adjusted using overlap weighting). For propensity score-weighted models (ATO), sandwich variance estimators were applied; for matched models, cluster-robust variance estimators were used to account for pair clustering.

Reporting both cause-specific Cox hazard ratios (HR) and Fine–Gray subdistribution hazard ratios (sHR) provides a complete and transparent characterization of both the underlying physiological susceptibility and the real-world absolute cumulative burden of non-fatal cardiac readmissions.

Supplementary Methods S1.4. Incremental Prognostic Value and Model Discrimination (Harrell's C-index)

To evaluate whether detecting non-ischaemic myocardial injury (NIMI) provides incremental prognostic information beyond established clinical risk factors and chronic comorbidities, model discrimination was evaluated using Harrell's concordance index (C-index) for censorship-adjusted survival data. Two hierarchical baseline risk models were constructed for primary outcomes (5-year all-cause mortality and MACE):

Model 1 (Classic Cardiovascular Risk Factors): Included age, sex, hypertension, diabetes mellitus, dyslipidaemia, active smoking, and established cardiovascular disease.

Model 2 (Comprehensive Baseline Risk): Expanded Model 1 by incorporating the Charlson Comorbidity Index, individual chronic comorbidities (chronic heart failure, previous stroke, peripheral vascular disease, chronic respiratory disease, chronic kidney disease), and baseline laboratory parameters (eGFR, haemoglobin, baseline glucose).

NIMI status was then added as an exposure variable to each baseline model. The incremental predictive performance was quantified by: Delta C-index: The absolute change in C-index after adding NIMI, with 95% confidence intervals calculated from 1,000 non-parametric bootstrap resamples. Likelihood Ratio (LR) Test: Comparing nested models (with vs. without NIMI) to evaluate statistical improvement in overall model fit. A statistically significant increase in the C-index\Delta C > 0, $p < 0.001$) and LR test ($p < 0.001$) confirms that NIMI provides independent, incremental predictive discrimination for long-term outcomes beyond comprehensive clinical profiling.

Supplementary Methods S1.5. Sensitivity Analyses (30-Day Landmark, E-Values, and COVID-19 Exclusion)

To assess the robustness of our primary findings and address potential sources of bias, three pre-planned sensitivity analyses were performed:

S1.5.1. 30-Day Landmark Analysis

To isolate chronic baseline vulnerability from acute presentation severity and eliminate potential contamination by early acute clinical deterioration, a landmark sensitivity analysis was conducted. The landmark point was set at 30 days following the index emergency department presentation. Patients who died or experienced a primary study endpoint (MACE or secondary readmissions) within the first 30 days were excluded. For all remaining 30-day survivors, follow-up was landmarked at day 30, and survival as well as competing-risk analyses (Fine–Gray subdistribution hazard models) were re-estimated for long-term outcomes (Supplementary Table S4, Supplementary Figures S4–S6). This confirmed whether the adverse long-term prognosis of NIMI persisted independently among individuals who survived the initial acute phase.

S1.5.2. E-Values for Unmeasured Confounding

To quantify the susceptibility of the observed treatment effect estimates to potential unmeasured confounding, E-values were calculated using the methodology proposed by VanderWeele and Ding. The E-value is defined as the minimum strength of association, on the risk ratio scale, that an unmeasured confounder would need to have with both the exposure (NIMI) and the outcome (e.g., 5-year mortality), conditional on the measured covariates, to fully explain away the observed hazard ratio. E-values and their lower 95% confidence bounds were computed for both the primary overlap-weighted (ATO) models and the 30-day landmark analyses across all study endpoints (Supplementary Table S5).

S1.5.3. Sensitivity Analysis Excluding COVID-19 Positive Patients

Given that recruitment coincided with a major surge of the SARS-CoV-2 pandemic (late 2020 to early 2021) and acute COVID-19 infection is a recognized cause of non-ischaemic myocardial injury, acute viral status was systematically recorded at index ED arrival. To ensure that pandemic-related acute mortality or viral myocardial injury did not drive the primary results, a sensitivity analysis was performed after completely excluding all patients with confirmed COVID-19 (n = 441). Propensity scores were fully re-estimated on the remaining uninfected cohort (n = 2,274), and overlap-weighted (ATO) hazard ratios were recalculated for all clinical outcomes (Supplementary Table S2).

Section S2. Supplementary Tables

Supplementary Table S1. Sample size, cohort composition, and covariate balance status across all eight propensity score models evaluated.

Propensity Score Model	Causal Estimand	Matching / Weighting Method	Control Group (n)	NIMI Group (n)	Balance Status (|SMD| < 0.10)
Unadjusted	None	None (Full Cohort)	2,461	254	Imbalanced
Basic Matched	None	1:1 Matching (Age + Sex)	254	254	Imbalanced*
Logistic Regression	ATO	Overlap Weighting	2,461 (Weighted)	254 (Weighted)	Perfect (SMD = 0.00)
Logistic Regression	ATT	1:1 Matching (Caliper 0.2)	251	251	Balanced
LASSO Regularization	ATO	Overlap Weighting	2,461 (Weighted)	254 (Weighted)	Perfect (SMD = 0.00)
LASSO Regularization	ATT	1:1 Matching (Caliper 0.2)	252	252	Balanced
Elastic-Net Regularization	ATO	Overlap Weighting	2,461 (Weighted)	254 (Weighted)	Perfect (SMD = 0.00)
Elastic-Net Regularization	ATT	1:1 Matching (Caliper 0.2)	251	251	Balanced

**Note:* Basic matched model only balances age and sex; significant chronic baseline comorbidity imbalances remain. Overlap weighting (ATO) models achieve exact mean balance (SMD = 0.00) of all included propensity score covariates by construction. ATO, average treatment effect in the overlap population; ATT, average treatment effect on the treated; LASSO, least absolute shrinkage and selection operator; NIMI, non-ischaemic myocardial injury; PS, propensity score; SMD, standardized mean difference.*

Supplementary Table S2. Sensitivity analysis excluding patients with confirmed COVID-19 infection.

Outcome	Analyzed Cohort (Non-COVID-19)	Overlap-Weighted HR (95% CI)
All-cause mortality	Uninfected cohort (*n* = 2,274)	1.72 (1.40–2.11)
Major adverse cardiovascular events (MACE)	Uninfected cohort (*n* = 2,274)	1.75 (1.44–2.12)
Heart failure readmissions	Uninfected cohort (*n* = 2,274)	1.60 (1.12–2.29)

A total of 441 patients with confirmed acute SARS-CoV-2 infection at index presentation (39/254 [15.4%] in the non-ischaemic myocardial injury group and 402/2,461 [16.3%] in the control group) were excluded. Propensity scores were fully re-estimated on the remaining uninfected cohort ($n = 2,274$) using chronic baseline covariates. Hazard ratios and 95% confidence intervals were calculated using marginal Cox proportional hazards regression with overlap weighting (ATO) and sandwich variance estimators. ATO, average treatment effect in the overlap population; CI, confidence interval; HF, heart failure; HR, hazard ratio; MACE, major adverse cardiovascular events; NIMI, non-ischaemic myocardial injury.

Supplementary Table S3. Incremental prognostic value of non-ischaemic myocardial injury over baseline risk models.

Outcome	Background model	C-index Base	C-index With NIMI	ΔC (95% CI)	LR *p*-value
All-cause mortality	Classic CV risk factors	0.7960	0.8056	0.0096 (0.0051–0.0147)	<0.001
All-cause mortality	Classic + chronic comorbidity	0.8243	0.8302	0.0058 (0.0029–0.0099)	<0.001
MACE	Classic CV risk factors	0.7967	0.8062	0.0095 (0.0056–0.0147)	<0.001
MACE	Classic + chronic comorbidity	0.8222	0.8280	0.0058 (0.0030–0.0089)	<0.001

Harrell's C-index was calculated from unweighted multivariable Cox models with and without NIMI, fitted to the same 2,708 patients. Seven patients with missing dyslipidaemia were excluded from this analysis after imputation of the chronic covariates. Classic cardiovascular risk factors were age, sex, hypertension, diabetes, dyslipidaemia and smoking. The expanded model also included Charlson index, previous MI, chronic HF, peripheral vascular disease, previous stroke, COPD, chronic kidney disease, eGFR, haemoglobin, glucose and malignancy.

C-index values describe discrimination in the study sample. Confidence intervals for ΔC were estimated from 500 bootstrap resamples, with both models refitted in each resample. The likelihood-ratio test compares models with and without NIMI. CI, confidence interval; COPD, chronic obstructive pulmonary disease; CV, cardiovascular; eGFR, estimated glomerular filtration rate; HF, heart failure; LR, likelihood ratio; MACE, major adverse cardiovascular events; MI, myocardial infarction; NIMI, non-ischaemic myocardial injury.

Supplementary Table S4. 30-day landmark sensitivity analysis clinical outcomes (cause-specific Cox HRs and Fine–Gray sHRs).

**Outcome**	**Control, *n***	**NIMI, *n***	**HR (95% CI)**	***p*-value**
All-cause mortality	2373	202	1.49 (1.19–1.86)	<0.001
MACE	2360	196	1.55 (1.26–1.92)	<0.001
MACE-2	2361	196	1.55 (1.25–1.92)	<0.001
HF readmissions	2361	196	1.58 (1.09–2.28)	0.016
Recurrent MI	2371	201	1.39 (0.57–3.40)	0.465

Hazard ratios and 95% confidence intervals were estimated by overlap-weighted Cox regression among patients alive, under observation and free of the relevant outcome beyond day 30 after ED presentation. Follow-up starts at day 30. Logistic propensity scores were refitted within each outcome-specific cohort using the chronic covariates. HRs for HF readmissions and recurrent MI are cause-specific. Counts are unweighted.

With death as a competing event, corresponding Fine–Gray estimates were sHR 1.39 (0.96–2.01), *p*=0.080, for HF readmissions, and sHR 1.24 (0.51–3.04), *p*=0.633, for recurrent MI.

HF, heart failure; HR, hazard ratio; MI, myocardial infarction; sHR, subdistribution hazard ratio. MACE comprises all-cause death, first HF readmission or first recurrent-MI readmission; MACE-2 comprises all-cause death or first HF readmission.

Supplementary Table S5. E-values for overall 5-year follow-up and 30-day landmark analyses across clinical outcomes.

Outcome	Full follow-up Estimate	Full follow-up CI limit	Day 30 Estimate	Day 30 CI limit
All-cause mortality	2.30	1.90	1.96	1.51
MACE	2.34	1.96	2.05	1.62
MACE-2	2.34	1.96	2.05	1.61
HF readmissions	2.66	1.60	2.53	1.40
Recurrent MI	2.60	1.00	2.14	1.00

E-values are shown for overlap-weighted Cox HRs and for the 95% confidence limit closest to 1. HF and MI results use cause-specific HRs. A CI-limit E-value of 1 indicates that the confidence interval includes 1. The EValue package's rare-outcome approximation was used for pooled event proportions below 15%, and its common-outcome HR conversion otherwise.

Section S3. Supplementary Figures

Supplementary Figure S1. Reverse Kaplan–Meier curve for overall cohort follow-up duration.

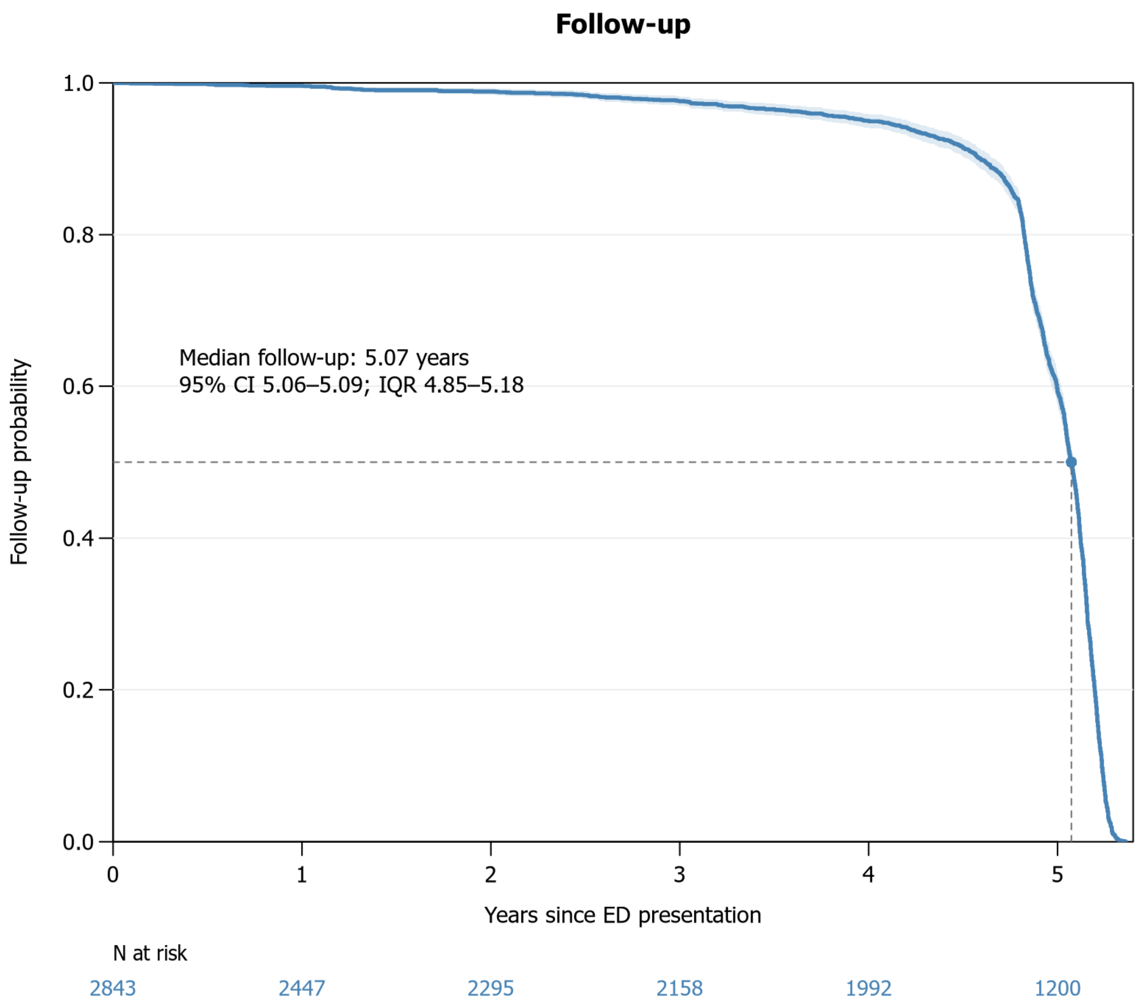

Follow-up distribution in the overall study cohort (*n* = 2,843). The solid line represents the reverse Kaplan–Meier estimate of observation duration, with shaded 95% confidence intervals. The dashed lines indicate the median follow-up of 5.07 years (95% CI 5.06–5.09; IQR 4.85–5.18). The table below displays the unweighted number of patients remaining under observation at each 1-year timepoint. CI, confidence interval; ED, emergency department; IQR, interquartile range.

Supplementary Figure S2. Fine–Gray subdistribution hazard ratios (sHR) forest plot for non-fatal cardiac readmissions

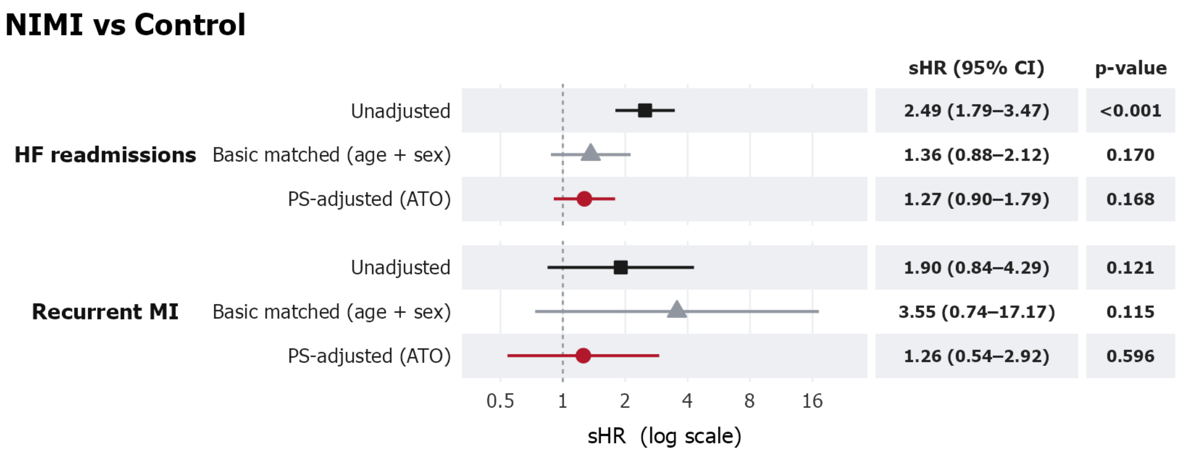

Subdistribution hazard ratios and 95% confidence intervals comparing non-ischaemic myocardial injury (NIMI) with the control group for first heart failure readmission and first recurrent myocardial infarction readmission across three levels of adjustment: unadjusted (black squares), basic matched using age and sex (grey triangles), and propensity score-adjusted using overlap weighting (red circles). All-cause mortality was treated as a competing risk. Models used the full recorded follow-up; *p*-values are from two-sided Fine–Gray proportional subdistribution hazard models with cluster-robust or sandwich variance estimators. ATO, average treatment effect in the overlap population; CI, confidence interval; HF, heart failure; MI, myocardial infarction; NIMI, non-ischaemic myocardial injury; PS, propensity score; sHR, subdistribution hazard ratio.

Supplementary Figure S3. Kaplan–Meier 5-year mortality survival curves across eight propensity score models.

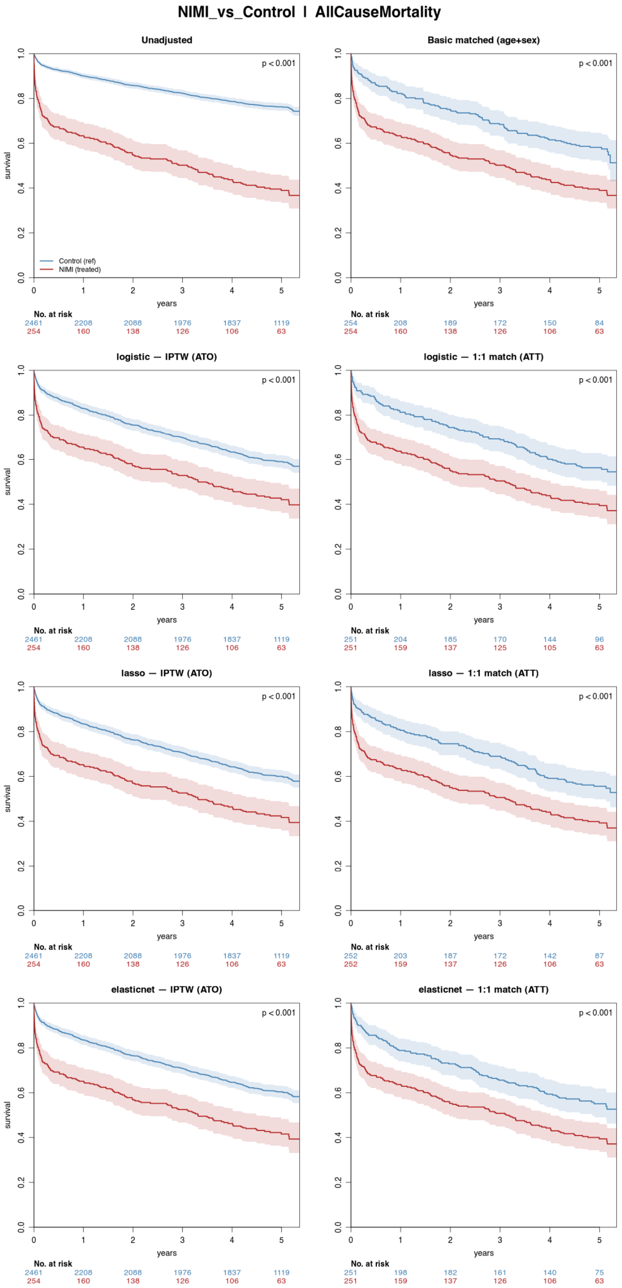

Cumulative survival curves over a five-year follow-up comparing patients with non-ischaemic myocardial injury (red lines) versus the control group (blue lines) across eight distinct statistical models, presented

in order from left to right and top to bottom: (1) unadjusted analysis (2,461 controls vs. 254 NIMI patients); (2) basic matched analysis for age and sex (*n* = 254 pairs); (3) standard logistic propensity score model using overlap weighting (ATO, *n* = 2,715); (4) standard logistic propensity score model using 1:1 nearest-neighbor matching (ATT, *n* = 251 pairs); (5) LASSO-regularized propensity score model using overlap weighting (ATO, *n* = 2,715); (6) LASSO-regularized propensity score model using 1:1 nearest-neighbor matching (ATT, *n* = 252 pairs); (7) Elastic-Net-regularized propensity score model using overlap weighting (ATO, *n* = 2,715); and (8) Elastic-Net-regularized propensity score model using 1:1 nearest-neighbor matching (ATT, *n* = 251 pairs). Shaded bands represent the 95% confidence intervals. In all eight models, patients with non-ischaemic myocardial injury experienced a significantly poorer long-term survival compared to the control group (all *p*-values < 0.001 estimated from marginal Cox proportional hazards models with robust variance estimators).

ATO, average treatment effect in the overlap population; ATT, average treatment effect on the treated; LASSO, least absolute shrinkage and selection operator; NIMI, non-ischaemic myocardial injury; PS, propensity score.

Supplementary Figure S4. 30-day landmark survival and cumulative incidence 3x3 grid.

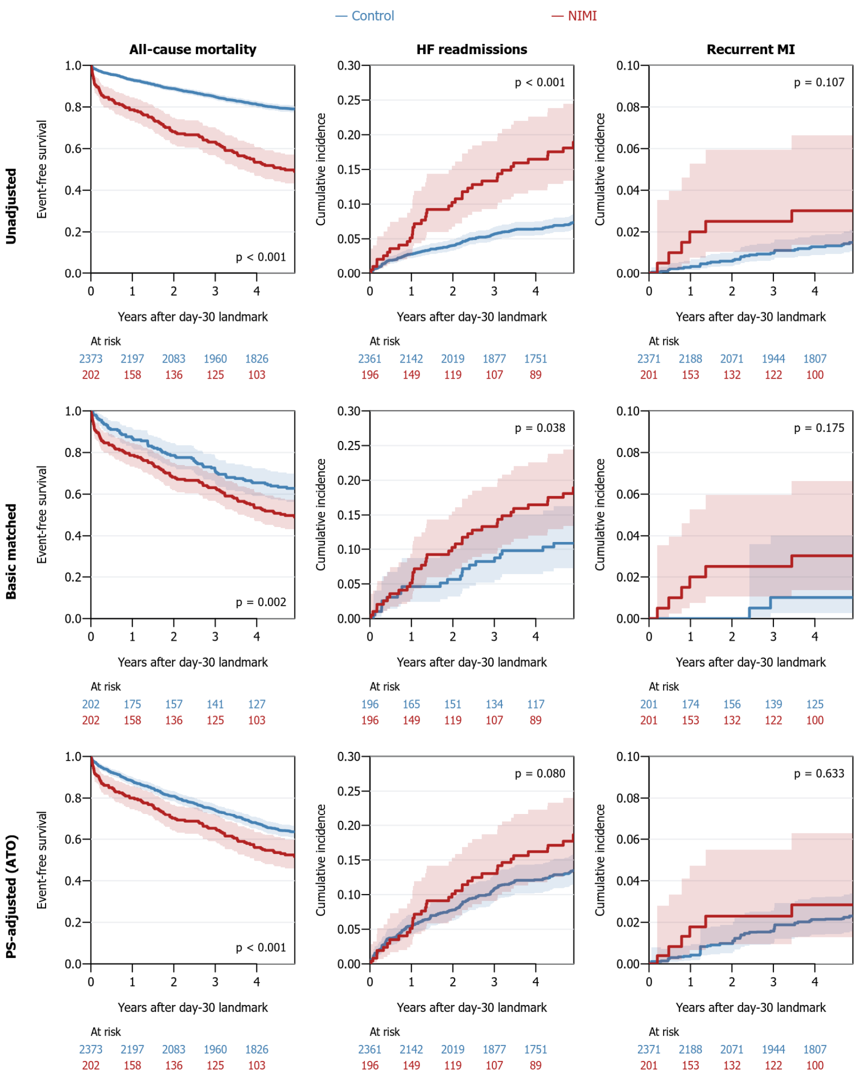

Landmark analysis conditioning on survival and absence of clinical events at day 30 following index ED presentation. Curves compare NIMI (red) with controls (blue). Columns show all-cause mortality (Kaplan–Meier survival), heart failure readmissions, and recurrent myocardial infarction readmissions (Aalen–Johansen cumulative incidence with death as a competing event). Rows represent unadjusted, basic matched, and overlap-weighted (ATO) analyses. Follow-up time starts at day 30. Shading represents 95% confidence intervals. Numbers at risk are unweighted. *P*-values are from Cox models for mortality and Fine–Gray models for readmissions. ATO, average treatment effect in the overlap population; NIMI, non-ischaemic myocardial injury; PS, propensity score.

Supplementary Figure S5. 30-day landmark cause-specific Cox hazard ratios forest plot.

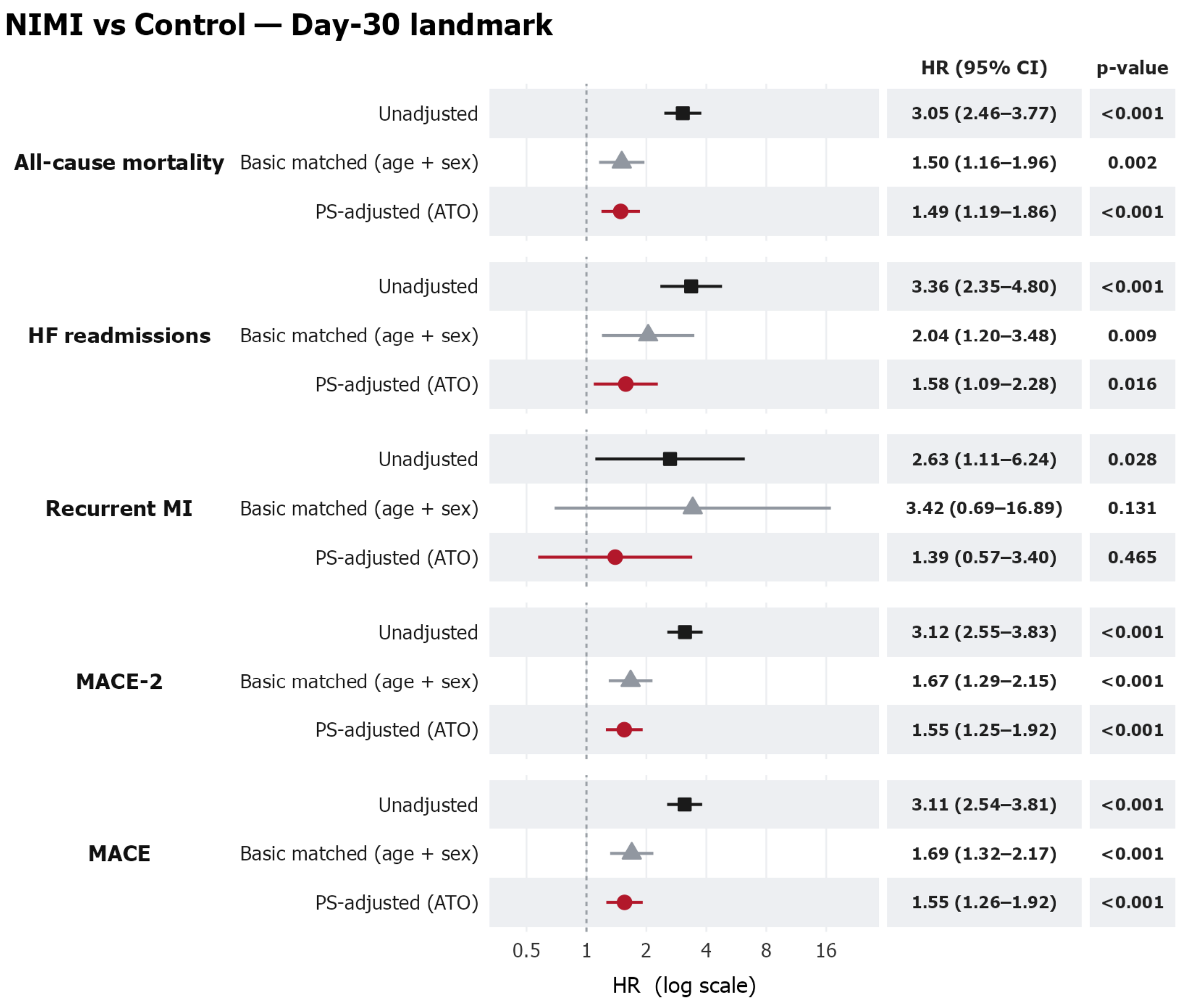

Cause-specific hazard ratios and 95% confidence intervals comparing NIMI with controls among 30-day survivors across study outcomes: all-cause mortality, heart failure readmissions, recurrent myocardial infarction readmissions, MACE, and MACE-2. Models evaluate unadjusted (black squares), basic matched (grey triangles), and overlap-weighted (red circles) Cox regressions with follow-up landmarked at day 30 post-presentation. ATO, average treatment effect in the overlap population; CI, confidence interval; HF, heart failure; HR, hazard ratio; MACE, major adverse cardiovascular events; MI, myocardial infarction; NIMI, non-ischaemic myocardial injury; PS, propensity score.

Supplementary Figure S6. 30-day landmark Fine–Gray subdistribution hazard ratios forest plot.

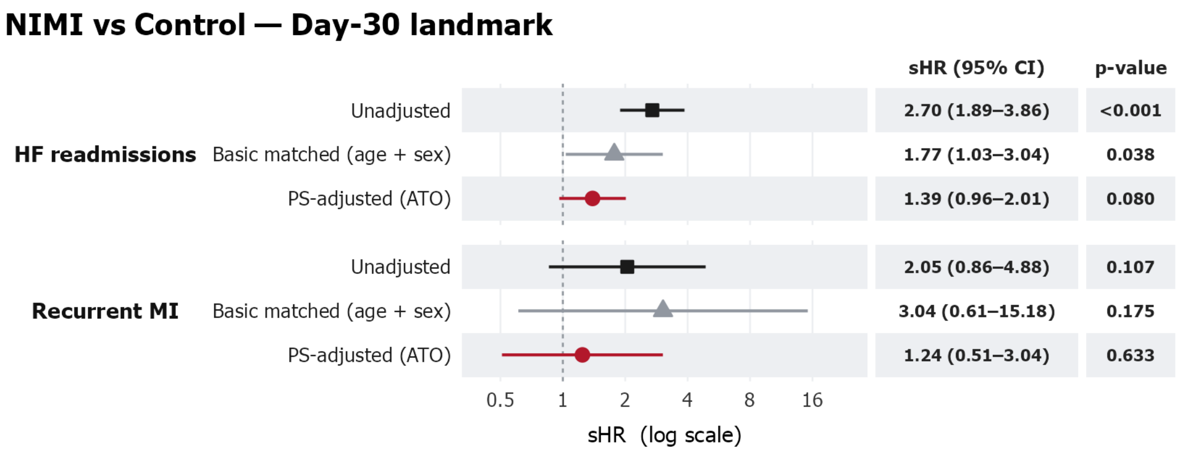

Subdistribution hazard ratios and 95% confidence intervals comparing NIMI with controls for non-fatal cardiac readmissions (heart failure and recurrent myocardial infarction) among 30-day survivors, treating death as a competing risk. Estimates are shown across unadjusted (black squares), basic matched (grey triangles), and overlap-weighted (red circles) Fine–Gray models. ATO, average treatment effect in the overlap population; CI, confidence interval; HF, heart failure; MI, myocardial infarction; NIMI, non-ischaemic myocardial injury; PS, propensity score; sHR, subdistribution hazard ratio.
